# Dual‐contrast micro‐CT enables cartilage lesion detection and tissue condition evaluation ex vivo

**DOI:** 10.1111/evj.13573

**Published:** 2022-03-30

**Authors:** Miitu K. M. Honkanen, Ali Mohammadi, Nikae C. R. te Moller, Mohammadhossein Ebrahimi, Wujun Xu, Saskia Plomp, Behdad Pouran, Vesa‐Pekka Lehto, Harold Brommer, P. René van Weeren, Rami K. Korhonen, Juha Töyräs, Janne T. A. Mäkelä

**Affiliations:** ^1^ 60650 Department of Applied Physics University of Eastern Finland Kuopio Finland; ^2^ 60650 Diagnostic Imaging Center Kuopio University Hospital Kuopio Finland; ^3^ Department of Clinical Sciences Faculty of Veterinary Medicine Utrecht University Utrecht The Netherlands; ^4^ 6370 Research Unit of Medical Imaging, Physics and Technology Faculty of Medicine University of Oulu Oulu Finland; ^5^ Department of Orthopedics University Medical Center Utrecht The Netherlands; ^6^ 60650 School of Information Technology and Electrical Engineering The University of Queensland Brisbane Australia; ^7^ 60650 Science Service Center Kuopio University Hospital Kuopio Finland

**Keywords:** articular cartilage, bismuth nanoparticles, contrast‐enhanced computed tomography, horse, ioxaglate, osteoarthritis

## Abstract

**Background:**

Post‐traumatic osteoarthritis is a frequent joint disease in the horse. Currently, equine medicine lacks effective methods to diagnose the severity of chondral defects after an injury.

**Objectives:**

To investigate the capability of dual‐contrast‐enhanced computed tomography (dual‐CECT) for detection of chondral lesions and evaluation of the severity of articular cartilage degeneration in the equine carpus ex vivo.

**Study design:**

Pre‐clinical experimental study.

**Methods:**

In nine Shetland ponies, blunt and sharp grooves were randomly created (in vivo) in the cartilage of radiocarpal and middle carpal joints. The contralateral joint served as control. The ponies were subjected to an 8‐week exercise protocol and euthanised 39 weeks after surgery. CECT scanning (ex vivo) of the joints was performed using a micro‐CT scanner 1 hour after an intra‐articular injection of a dual‐contrast agent. The dual‐contrast agent consisted of ioxaglate (negatively charged, *q* = −1) and bismuth nanoparticles (BiNPs, *q* = 0, diameter ≈ 0.2 µm). CECT results were compared to histological cartilage proteoglycan content maps acquired using digital densitometry.

**Results:**

BiNPs enabled prolonged visual detection of both groove types as they are too large to diffuse into the cartilage. Furthermore, proportional ioxaglate diffusion inside the tissue allowed differentiation between the lesion and ungrooved articular cartilage (3 mm from the lesion and contralateral joint). The mean ioxaglate partition in the lesion was 19 percentage points higher (*P* < 0.001) when compared with the contralateral joint. The digital densitometry and the dual‐contrast CECT findings showed good subjective visual agreement.

**Main limitations:**

Ex vivo study protocol and a low number of investigated joints.

**Conclusions:**

The dual‐CECT methodology, used in this study for the first time to image whole equine joints, is capable of effective lesion detection and simultaneous evaluation of the condition of the articular cartilage.

## 1 INTRODUCTION

Osteoarthritis (OA) is a frequent joint disease in the horse.^1^ The disease may be initiated by joint trauma that produces cartilage lesions, which may further deteriorate and culminate in the development of post‐traumatic OA,^2^, ^3^ with accompanying clinical signs such as lameness. Early and accurate detection of cartilage damage is key for successful early intervention, allowing for effective treatment and prevention of further damage.^4^, ^5^ Animal models provide an important contribution to OA research.^6^, ^7^ One of the previously used models is the groove model. This is a model that induces trauma of the hyaline, non‐calcified cartilage layer surgically and is used for studying the progression of degenerative changes within a joint.^8^, ^9^

Radiography, ultrasonography, arthroscopy, computed tomography (CT) and magnetic resonance imaging (MRI) are medical imaging techniques that have been used for articular cartilage imaging.^10^ Magnetic resonance imaging is an excellent imaging modality for imaging soft tissues with high water content, like articular cartilage. However, low magnetic field MRI (≤0.3 tesla), as mostly used in equine practice, cannot detect small cartilage lesions.^11^, ^12^, ^13^ Plain radiography and CT allow for faster imaging and significantly better image fidelity for small objects. But, due to relatively similar X‐ray attenuation of synovial fluid and cartilage, plain radiography and CT are not able to distinguish cartilage and its lesions from synovial fluid.^10^

Contrast agents enhance the distinction between synovial fluid and articular cartilage in MRI and CT images.^14^ Anionic (negatively charged) contrast agents, eg ioxaglate, are commonly used for intra‐articular contrast‐enhanced CT (CECT).^15^ The resolution of modern clinical CT devices in combination with contrast media enables accurate evaluation of cartilage thickness and qualitative assessment of cartilage condition.^16^, ^17^, ^18^ After the contrast agent injection in vivo, optimal timing allows scanning with maximum deposition of agent inside cartilage, before it clears from the synovial space. The distribution of ioxaglate within cartilage is inversely proportional to the proteoglycan (PG) distribution in the extracellular matrix (related to the negative fixed charge density).^19^, ^20^ A superficial collagen disruption, decrease in PG content and the resulting increase in water content are early signs of osteoarthritic degeneration.^21^ These changes decrease the fixed charge density and increase the tissue permeability, subsequently increasing the diffusion of negatively charged contrast agents into cartilage.^22^

Contrast‐enhanced CT with ioxaglate requires two image acquisitions: immediately and at a delayed time point after contrast agent injection.^10^, ^17^ The immediate scan evaluates the articulating surface when there is still a good contrast between synovial fluid and articular cartilage. As the contrast agent diffuses into the cartilage layer, this contrast diminishes. The second scan enables quantitative evaluation of the cartilage condition.^17^

A bipartite contrast agent, comprising ioxaglate and neutral bismuth nanoparticles (BiNPs) which are large enough to remain in the joint space, allows for characterisation of the tissue using only one scan, without the need for complex co‐registration of separate scans.^23^ In this study, we aim to investigate the capability of this technique used previously on osteochondral samples to visualise surgically induced blunt and sharp cartilage defects in equine carpi ex vivo. We hypothesise that in addition to visualising the grooves with the dual‐contrast technique, we can simultaneously evaluate the PG content of the cartilage.

## 2 MATERIALS AND METHODS

### 2.1 Animal selection and preparation

Nine female Shetland ponies (aged 6.8 ± 2.6 years; bodyweight was 203 ± 15.3 kg) were included. As no prior contrast agent (ioxaglate) partition data for whole equine joints were available, the number of ponies was based on a power analysis (power 0.90 and *P* < 0.05) pertaining to the macroscopic and microscopic scores (OARSI) in previous studies.^8^, ^9^, ^24^, ^25^, ^26^ Retrospectively, based on the ioxaglate partitions as determined in this study, the statistical power was found to range from 0.83 to 0.94, depending on the location and the groove type. Average cartilage thickness for the ponies was 0.501 ± 0.101 mm (defined previously from micro‐CT images at six locations adjacent to grooves). For comparison, the cartilage thicknesses in the carpal joint of trained and untrained Thoroughbred horses has been reported to be 0.7 and 0.6 mm, respectively.^27^ Prior to the experiments, the ponies were in good health and did not suffer from clinically visible lameness or joint injuries.

**FIGURE 1:**
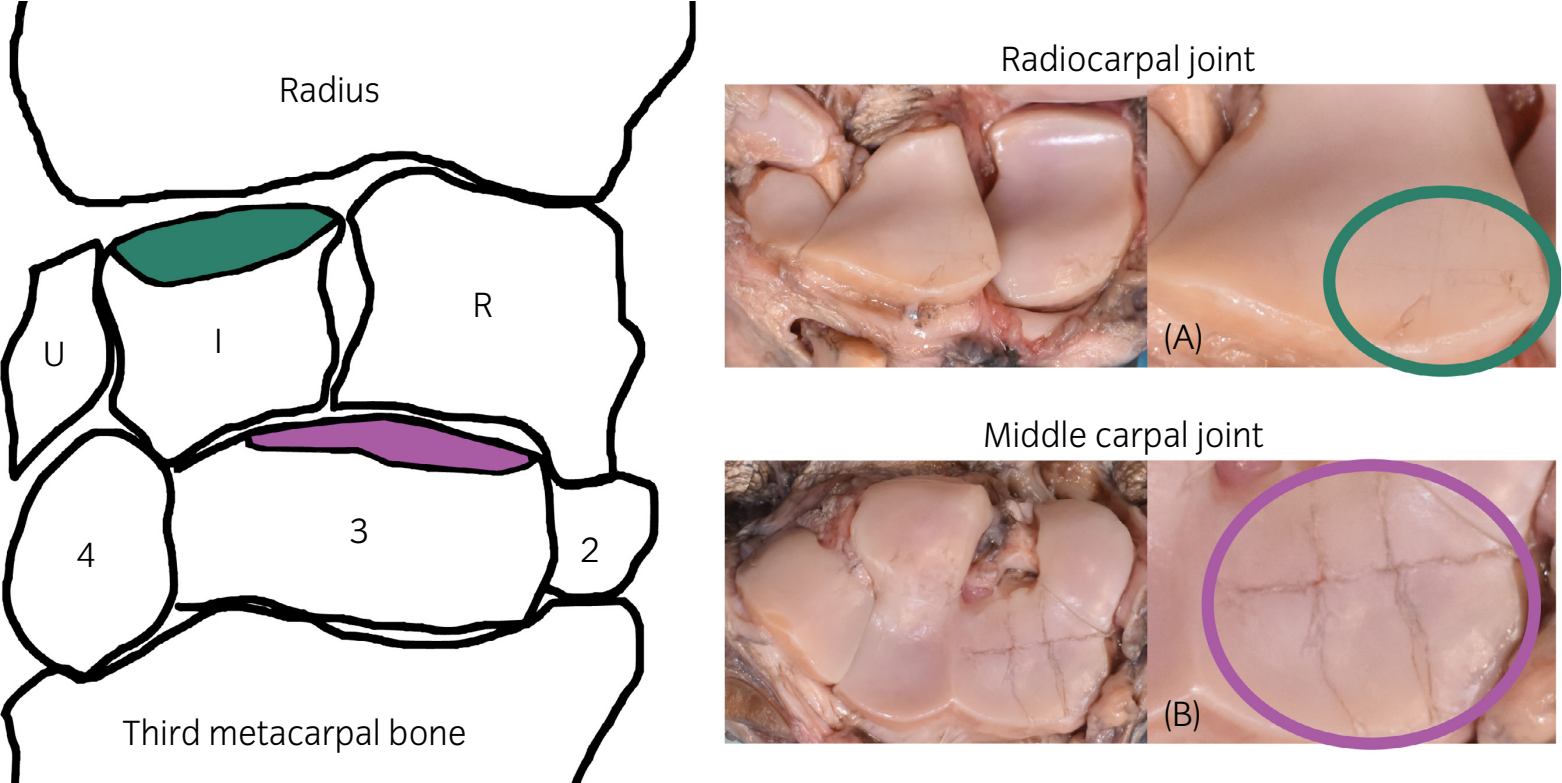
Illustration of the equine carpus anatomy and photographs of the articular cartilage surfaces of the radiocarpal and middle carpal joints. Bones are marked as U = ulnar carpal bone, I = intermediate carpal bone, R = radial carpal bone, 4 = fourth carpal, 3 = third carpal bone, and 2 = second carpal bone. Locations for the grooves at the proximal surface of I (A, marked with green) and 3 (B, marked with purple) are highlighted. Sharp (A) and blunt (B) grooves were randomly made in the radiocarpal and middle carpal joints

All surgeries were performed by a board‐certified equine surgeon under general anaesthesia with isoflurane and continuous rate infusion with detomidine (Domosedan^®^, Vetoquinol BV, 0.01 mg/kg/h). For all animals, blunt and sharp grooves were created in one randomly selected front limb (Figure 1) via an open surgical approach to the joint using an incision as small as possible needed for exposure of the articular cartilage (mini‐arthrotomy).^25^ Blunt or sharp grooves were randomly made in the radial facet of the third carpal bone and in the intermediate carpal bone (dorsoproximal surface). Blunt grooves were created with an arthroscopic probe with a sharpened tip and sharp grooves with a surgical blade (Beaver Mini‐Blade^®^, MFID: 376400) mounted in a custom‐made device that limited penetration depth to 400 μm. The contralateral joints were sham‐operated without causing any damage to the cartilage surfaces and served as control joints. After 3 weeks of complete box‐rest, the ponies started a controlled exercise programme on a treadmill (Mustang 200, Kagra AG). After 26 weeks, they were given free exercise at pasture until the end of the study. The ponies were euthanised 39 weeks after surgery, and the carpal joints were harvested and stored at −20°C until the micro‐CT imaging. In vivo monitoring including arthroscopic near infrared imaging, radiographs, synovial biopsies, and synovial fluid samples during the 9‐month‐follow‐up period was conducted. A more detailed description of the surgical procedures, in vivo measurements, and exercise programme have been reported by te Moller et al and Sarin et al,^25^, ^28^ and this study was a component of a previously reported project.^25^

### 2.2 Contrast agent preparation

Bismuth nanoparticles (BiNPs) were prepared according to ‘targeted pyrolysis’ approach, developed and published by our group.^29^ Briefly, bismuth oxide powder (Sigma‐Aldrich) was ball‐milled in H_2_O for 2 hours at a speed of 1000 rpm. Next, the milled bismuth oxide was washed first with ethanol and then mixed with polyethylene glycol–silane (PEG–silane, 0.5 kDa, Gelest). Ethanol was evaporated at 80°C with a N_2_ flow for 20 minutes. The bismuth oxide was reduced into elemental BiNPs and subsequently functionalised with PEG at 230°C in one‐pot ‘targeted pyrolysis’ reaction process. BiNPs were stored in ethanol until the micro‐CT measurements. Before mixing the contrast agent solution, the ethanol was separated out by centrifuging (10 000 rpm) the solution for 5 minutes. Ultrasound was used to disperse the BiNPs in distilled water. This centrifuging‐dispersion process was repeated to ensure that ethanol was properly removed from the BiNP solution. Right before the intra‐articular injection, the BiNPs were mixed with ioxaglate (M = 1269 g/mol, Hexabrix, Mallinckrodt Inc) to create a dual‐contrast agent. Concentrations were 160 mg I/mL and 20 mg/mL for ioxaglate, and BiNP, respectively. Osmolality was adjusted to that of healthy synovial fluid: 400 mOsm/kg.^30^, ^31^

### 2.3 Contrast‐enhanced micro‐CT

**FIGURE 2:**
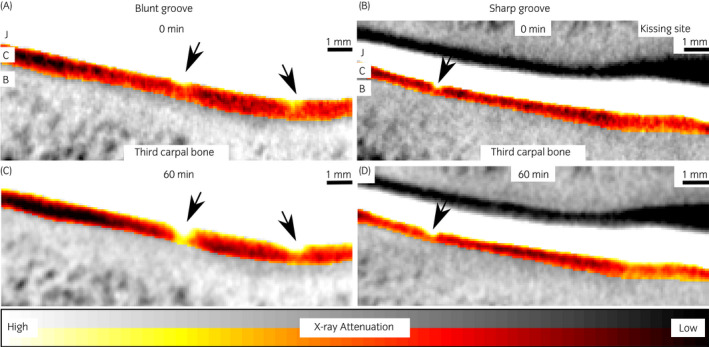
Coronal computed tomography images of blunt and sharp grooves immediately (0 minutes) and 60 minutes after dual‐contrast agent (ioxaglate + bismuth nanoparticles, BiNPs) injection procedure for different individuals. BiNPs that remain in the joint cavity (J) enable cartilage (C) surface detection at the delayed time point with both lesion types. Bone (B) is marked in the 0 minutes images, and all grooves are marked with black arrows

Joints were thawed overnight at 4°C. Dual‐contrast agent was injected into the radiocarpal and middle carpal joints (10 mL each). After injection, the joints were cyclically flexed and extended for 2 minutes with the full range of motion to ensure dispersion of the contrast agent into the entire joint cavity. A baseline (*t* = 0 minute) image of the joint was acquired with a cone beam micro‐CT scanner (Quantum FX^®^, Perkin Elmer) in the axial plane using 90 kV tube voltage and 200 mAs (Figure 2). Second image acquisition was conducted 60 minutes after the injection procedure. The imaging parameters were: field of view = 40 × 40 mm^2^, isotropic voxel size = 80 µm and acquisition time = 120 seconds. Three‐dimensional reconstruction was conducted with Quantum FX software. The joints were kept at room temperature between the scans. Additionally, due to the size of the joints and spatial constraints, water and BiNP (20 mg/mL) phantoms were imaged separately to define the ioxaglate partition within the cartilage.

### 2.4 Data analysis

To allow visual comparison (Figure 2) between the two imaging time points, delayed arthrography (*t* = 60 minutes) image stacks were co‐registered separately for each bone (third carpal bone and intermediate carpal bone) with the first (*t* = 0 minute) time point image stacks using MIM software (version 6.9.4, MIM Software Inc.). Next, cartilage surfaces were manually segmented in the axial plane with the MIM software, after which the segmentations were finalised in coronal plane using 3D Slicer software (version 4.8.1, Kitware, Inc, Brigham and Women's Hospital).^32^, ^33^

To reduce noise, the CT images that were used for analysis were an average of six consecutive slices (total thickness = 0.48 mm). To evaluate the state of cartilage degeneration caused by the grooves, average ioxaglate partition, ie the relative concentration compared with the concentration in the joint cavity, was calculated for the lesion (volume of interest (VOI): 0.48 mm × 0.48 mm × cartilage thickness, including the groove). Analysis was also done for tissue 3 mm away from the groove and at the location corresponding to the groove in the contralateral joint. These ungrooved VOIs (1.04 mm × 0.48 mm × cartilage thickness) were enlarged to reduce noise. One radiocarpal joint having sharp grooves was left out of the analysis due to a big air bubble in the joint cavity having prevented contrast agent diffusion into the cartilage. Finally, nine joints having blunt grooves and eight joints having sharp grooves were included in this study.

Depth‐dependent ioxaglate partition profiles were calculated as follows: cartilage tissue was cropped using manual segmentation and a threshold (the null point of the derivative) at the cartilage–bone interface. The natural curvature of the cartilage surface and cartilage–bone interface was removed by interpolating the X‐ray attenuation profiles within the VOI to the same length. Next these attenuation profiles within the VOI were averaged to get one profile over the full thickness of the hyaline cartilage layer, from the calcified cartilage to the articulating surface. Finally, to establish the attenuation of ioxaglate within cartilage, estimated X‐ray attenuation of a native (ie non‐contrast‐enhanced) cartilage (1.05 × the attenuation of water) was subtracted from the acquired attenuation profile. The X‐ray attenuation of the separate BiNP suspension was subtracted from the attenuation of the dual‐contrast agent when defining the attenuation of plain ioxaglate in the joint cavity. This was then used to calculate the ioxaglate partition inside the cartilage.

Depth‐dependent ioxaglate profiles were determined for the lesion, 3 mm away from the groove, and VOI in the contralateral joint. Statistical significance of differences in ioxaglate partition and optical density (OD) between different locations was evaluated using a Kruskal‐Wallis test. The inspection was done separately for the different grooves, but also with the blunt and sharp groups combined. The level of statistical significance was set at *P* < 0.05. Bonferroni correction was used to reduce type I error for multiple comparisons. The statistical analyses were conducted using SPSS (v. 27 SPSS Inc, IBM Company).

### 2.5 Digital densitometry

After the micro‐CT imaging, the grooved sites along with the contralateral controls were harvested using an oscillating saw (multitool PMF 220CE, Bosch) and stored in −20°C. The samples were 12 mm wide rectangular osteochondral samples; 6 mm from the centre of the groove that ran in latero‐medial direction, towards the dorsal and the palmar side. After thawing, the samples were analysed using reference methods (micro‐CT imaging and mechanical indentation; not employed in this study)^25^ and were finally fixated in 10% formalin and decalcified in 0.5 M EDTA (prod. 20296.360, VWR) at pH 7.0 for 10 weeks. After the decalcification, two parts (3 mm in width) were cut at 3 mm from the horizontal groove on both sides and embedded in paraffin (illustration available in te Moller et al^25^). Afterwards, three adjacent 5 μm thick sections were cut with a microtome and stained with Safranin‐O/Fast‐green (SOFG).

Digital densitometry (DD) was used to quantify the distribution of the PG content, and to compare the PG distribution with the ioxaglate distribution, determined from the micro‐CT images. DD images were acquired with a light microscope (Nikon Microphot‐FXA, Nikon Co.) equipped with a CCD camera (ORCA‐ER, Hamamatsu photonics KK), using 4× magnification (pixel size = 1.4 μm). The OD images were calibrated against neutral density filters (OD values: 0.0, 0.3, 0.6, 1.0, 1.3, 1.6, 2.0, 2.3, 2.6 and 3.0, Schott).^6^, ^34^ The matching of ioxaglate concentration and OD profiles was done based on the distance from the groove.

## 3 RESULTS

**FIGURE 3:**
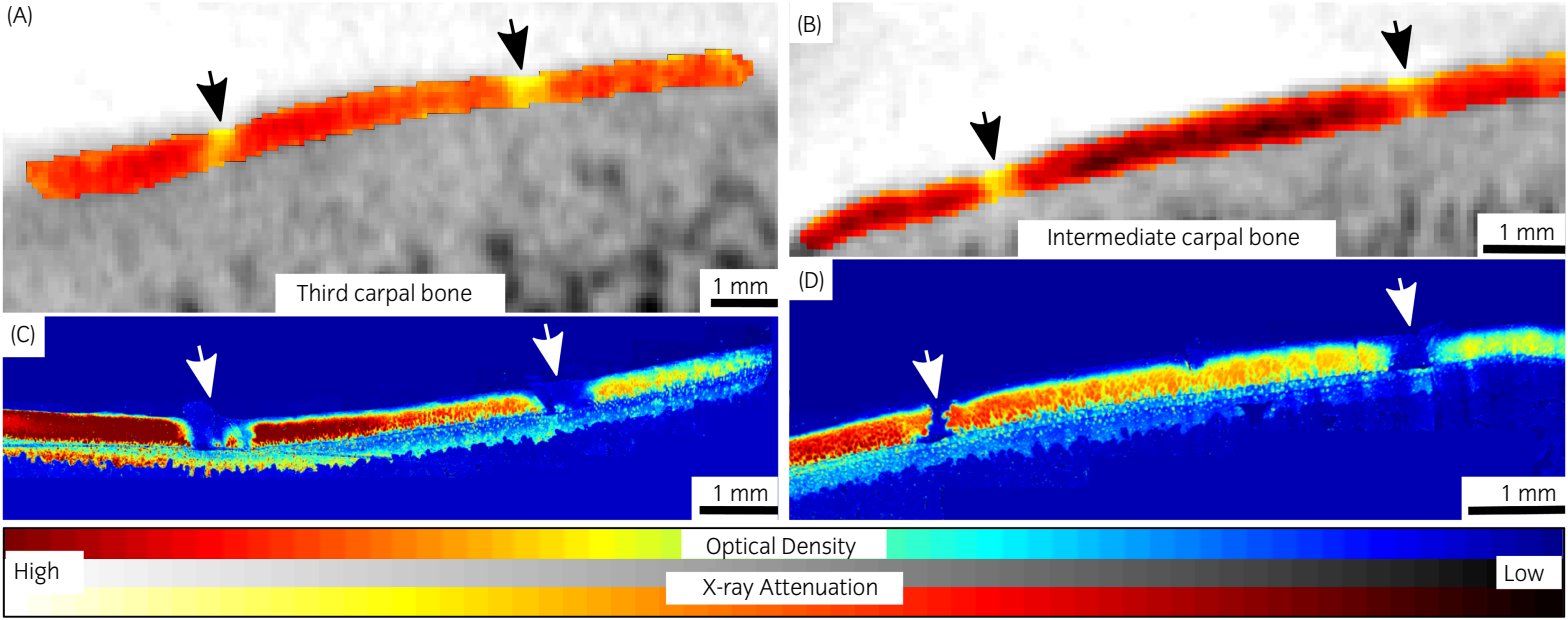
Computed tomography (CT) (A and B) and optical density (OD)‐maps (C and D) of two ponies having blunt grooves (black and white arrows in CT‐ and OD‐maps, respectively) on the articular surfaces of the third carpal bone and the intermediate carpal bone. The grooves are well detectable, and higher ioxaglate uptake near the lesion is visible in the CT images at the 60 minutes time point

**FIGURE 4:**
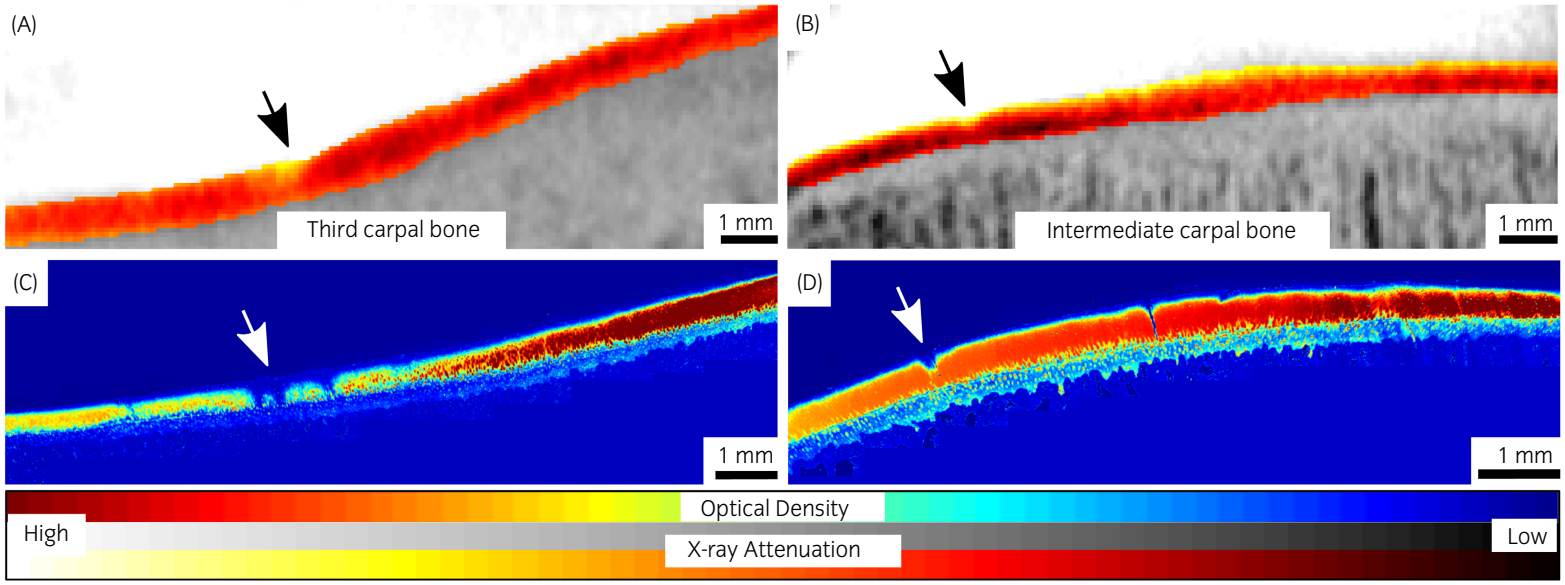
Computed tomography (A and B) and optical density maps (C and D) of two ponies having sharp grooves (black and white arrows) on the articular surfaces of the third carpal bone and the intermediate carpal bone. Computed tomography maps, acquired at the 60 minutes time point, show a degeneration‐related increase in ioxaglate diffusion into the cartilage at the grooved region and immediately adjacent to this. Similarly, proteoglycan loss is visible in the optical density maps

**FIGURE 5:**
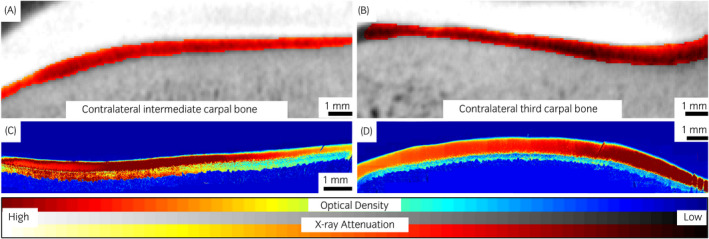
Computed tomography (A and B) and optical density maps (C and D) for contralateral (control) joints with intact articular cartilage surfaces of the third carpal bone and the intermediate carpal bone for two individual ponies. Computed tomography imaging was conducted at 60 minutes after the contrast agent injection procedure

The dual‐contrast enhanced micro‐CT images reflected well the different conditions of the defects and healthy cartilage, showing a smooth regular surface for control tissue (contralateral joint), and irregular clefts with increased attenuation at the grooved locations (Figures 3, 4, 5). The BiNPs, too large to penetrate the tissue, highlighted the topography of the articulating surface. The blunt grooves were particularly well visible (Figures 2 and 3), but the thinner sharp grooves were also visually distinguishable on the micro‐CT images (Figures 2 and 4). Increased attenuation deeper in the tissue pointed to enhanced ioxaglate penetration and build‐up of the contrast agent, as a result of peri‐defect deterioration (Figures 3 and 4). This was not observed for healthy cartilage; for the contralateral healthy tissue, the maps of the ioxaglate attenuation displayed homogeneity (Figure 5).

**FIGURE 6:**
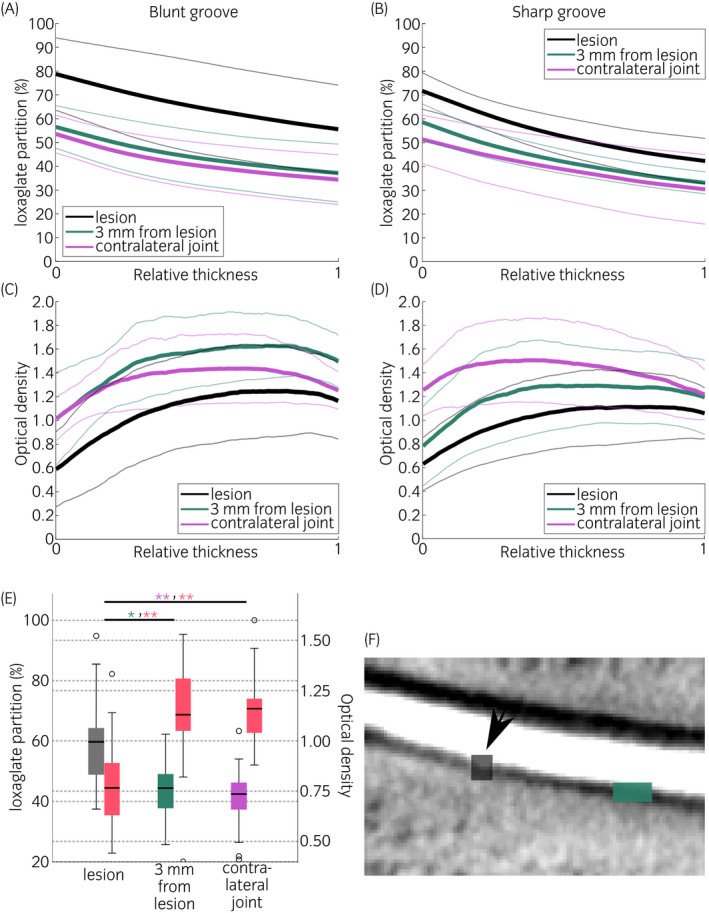
Mean depth‐dependent ioxaglate partition (A and B, thick lines) and mean depth‐dependent optical density (OD) profiles (C and D, thick lines) of three locations in blunt (n = 9, A and C) and sharp‐grooved joints (n = 8, B and D) and their contralateral controls. Ioxaglate partition and OD were determined at two locations on the grooved site: lesion (black), 3 mm from the lesion (green), and at one location in the contralateral joint (purple), matching the lesion location. On the horizontal axis, 0 represents the articulating surface and 1 the articular cartilage–subchondral bone interface. Thinner lines indicate standard deviation. Ioxaglate partition and OD (red) in full cartilage thickness (E). Boxplots represent the contrast agent partition/OD (±interquartile ranges) in grooved joints (sharp and blunt grooves together) for the lesion, 3 mm from the lesion, and in the contralateral joint. Median partitions are marked with thick line inside the box, maximum and minimum partitions with (‐) and outliers with (o). Statistically significant differences between regions of interest have been marked with * (*P* < 0.01) and ** (*P* ≤ 0.001). Example of the VOI locations (F): lesion marked with black square and arrow, and 3 mm from the lesion with green rectangle

The resolution of the micro‐CT system enabled depth‐dependent profiling of the tissue with good accuracy (Figure 6). Mean ioxaglate partition for full thickness cartilage was 19 (*P* < 0.001) and 16 (*P* = 0.006) percentage points higher for both types of lesions, compared to the contralateral joint and 3 mm away from the groove, respectively. The same difference between lesion and contralateral joint for the blunt groove was 24 (*P* = 0.041) percentage points and for the sharp groove was 15 (*P* = 0.019) percentage points (Table 1). OD maps exhibited visual erosion and a decrease in staining (ie estimate of cartilage fixed charge density) at grooved locations (Figures 3 and 4), similar to the micro‐CT images. Significant difference in OD between lesion and contralateral joint was observed for blunt (*P* = 0.035) and sharp (*P* = 0.033) grooves (Table 1). In addition, significant (*P* = 0.002) difference in OD between the lesion and 3 mm away from the lesion was found for the blunt groove.

**TABLE 1:** Mean ioxaglate partitions (%) and standard deviations in full thickness cartilage at three different locations (lesion, 3 mm away from the lesion, and matching location [for lesion] in the contralateral joint), for blunt and sharp grooves. Mean optical densities and standard deviations for grooves and ungrooved tissues.

Region of interest	Blunt groove		Sharp groove	
	Ioxaglate partition (%)	Optical density	Ioxaglate partition (%)	Optical density
Lesion	66 ± 18	0.7 ± 0.2	54 ± 7	0.8 ± 0.2
3 mm away from lesion	45 ± 11	1.2 ± 0.2^**^	43 ± 5	1.0 ± 0.3
Contralateral	42 ± 9^*^	1.1 ± 0.2^*^	39 ± 12^*^	1.2 ± 0.2^*^

Statistically significant differences between lesion and ungrooved tissues have been marked with * (*P* < 0.05) and ** (*P* < 0.01).

## 4 DISCUSSION

In the current study, delayed dual‐contrast micro‐CT was used, for the first time, for whole equine joints ex vivo. With a single scan, the method enables simultaneous visualisation of the articulating surfaces and quantification of the tissue degenerative state. We showed that the surgically made grooves can be easily differentiated from the ungrooved cartilage, and the cartilage condition around the groove can be visually evaluated from the differing ioxaglate uptake.

The blunt grooves were clearly distinguishable on the CT images (Figure 3). The BiNPs highlighted very effectively the relatively large cavities of the blunt grooves. As the X‐ray absorption properties of articular cartilage are very close to those of synovial fluid, without using the BiNPs the articulating surface would have been blurry, making segmentation and inspection of the surface condition difficult or impossible.^17^ Furthermore, ioxaglate that diffuses into the cartilage does not hinder the visualisation of the interface between the tissue and the joint cavity when combined with the BiNPs (Figure 2). The sharp grooves were visually distinguishable even though OD‐maps (Figure 4) showed that the tissue surface was relatively smooth and fairly regular. te Moller et al reported significant differences between the blunt and sharp grooves in OARSI microscopy scores (*P* = 0.007) and in fixed charge density (*P =* 0.006), when cartilage condition around the grooves was inspected. Albeit, the overall degeneration remained moderate.^25^ The present dual‐contrast CECT method or OD with fixed VOIs were not able to differentiate between the lesion types. In future, use of similar grading scales as introduced in MRI studies (such as the WORMS scoring^35^) could help with this issue.

The groove model chosen for this study differs from naturally developing idiopathic OA. However, the blunt and sharp grooves have been used to study OA development^8^, ^9^, ^36^, ^37^ and these lesions resemble defects that could occur on the cartilage surface after injury, before they lead to post‐traumatic OA. In lesion area, cartilage deterioration caused by the blunt and sharp grooves showed as increased X‐ray attenuation, as a result of the larger amount of ioxaglate inside the tissue due to the increased permeability and decreased PG content, as confirmed in the OD‐maps (Figures 3, 4, 6, and Table 1). This objective, numerical information might benefit surgeons, who need to assess the extent of trauma and decide whether there is a need for surgical intervention. Ioxaglate partition was 60 ± 15% in blunt and sharp grooves, which was significantly (*P* < 0.01) higher than the partition in ungrooved tissues [3 mm away from lesion (44 ± 9%) and contralateral joint (41 ± 11%)]. Similarly, significant (*P =* 0.001) difference in OD was observed between lesion (0.79 ± 0.23) and ungrooved tissues (1.13 ± 0.23 for 3 mm away from the lesion and 1.16 ± 0.19 for contralateral joint) when sharp and blunt grooves were inspected together.

The *t* = 0 minute time point background scan was performed just for visual comparison purposes. The dual‐contrast method obviates the need for this scan, as the BiNP nanoparticles maintain a good contrast at the delayed time point (*t* = 60 minutes, Figure 2) when the ioxaglate has diffused into the cartilage, allowing for the quantitative assessment of the tissue's condition. The high X‐ray attenuation of the joint cavity, occupied by the dual‐contrast agent (1667 HU at the *t* = 60 minutes time point), compared with cartilage (1054 HU in lesion and 747 HU in ungrooved tissue) allows future studies to use automated cartilage segmentation. The delayed time point (60 minutes) was based on a previous diffusion study with human knee joints (in vivo) as this was the time point when maximum concentration of ioxaglate was reached.^17^ We chose to concentrate on the clinical relevancy even though the diffusion conditions between the studies differ (equine carpal joint, ex vivo and room temperature). Studying the effect of time on the diffusion was not the scope of this study, as it has been done before.^17^, ^38^

The present study is not without limitations. The sample pool was relatively small, having eight and nine joints in sharp and blunt groove groups, respectively. The sham‐operated contralateral control joints were not entirely free from cartilage lesions, which were most probably caused by repeated arthrocentesis during the 9 months follow‐up period after surgery.^25^ Histological sections were cut perpendicular to the two grooves that ran in dorsopalmar direction. This orientation could have differed from the orientation in the CT segments and, thus, total parallelism between the DD and micro‐CT slices could not be guaranteed. Grooved VOIs were smaller than the ungrooved VOIs, potentially causing imprecision due to altered variance. Injection of contrast media resulted in small air bubbles in the joint space in a number of joints. This could potentially have affected the diffusion, co‐registration, segmentation, and, ultimately, analysis. One joint (with sharp grooves) which featured a big air bubble was excluded. The phantoms were not included in the image acquisitions (due to lack of space), and possible minor attenuation fluctuation between the scans originating from the microCT could not be evaluated. However, the air attenuation values were checked during the scanning procedure and found to be at the same level. Furthermore, scanning whole joints of large animals leaves the results vulnerable to the beam hardening artifact, which occurs when photons of lower photon energy levels are attenuated more and can create an error in determining the contrast agent partition value. Thus, this can lead to variation in the partition values regardless of the cartilage condition and subsequently, could lead to misinterpretation of the results. Unfortunately, it was impossible to determine the extent to which beam hardening influences the achieved results. Finally, when calculating the contrast agent partition, the sample‐specific variation in X‐ray attenuation in native cartilage was not taken into account. Instead, we used 1.05 times the water X‐ray absorption value when calculating ioxaglate partition inside the cartilage, as we tried to simulate the clinical situation in which only one delayed imaging time point is available. Ultimately, the main purpose of this study was to visualise the lesions and the related cartilage tissue deterioration.

Refining the contrast agent product and fine‐tuning the clinical scan settings were not within the scope of this project. The final clinical CECT technique may in the future rely on contrast agent formulations differing from the one applied here. The feasibility of clinical use of the BiNPs will be studied in future projects. One proposed alternative is to substitute ioxaglate with a cationic agent that diffuses into cartilage proportionally to the PG content.^39^, ^40^ Moreover, the diagnostic sensitivity of the cationic agent can be further enhanced with a non‐ionic compound, nullifying the variation in the diffusion caused by changes in tissue water content and permeability.^41^, ^42^ Our results form a good basis to proceed on this path, as these show clear differences in ioxaglate partition values between lesions and healthy tissue, even though cationic agent have exhibited stronger correlation with PGs (*R* ^2^ = 0.83) than negatively charged agents (*R* ^2^ = 0.20).^19^

In conclusion, in the current study the dual‐contrast method was, for the first time, applied to evaluate the cartilage condition in equine joints. Simultaneous detection of lesion width and depth and assessment of the condition of the surrounding tissue proved possible with only one delayed imaging time point. To further optimise the technique, contrast agent circulation within and out of the joint space should be evaluated in future in vivo studies with novel standing CT scanners. Based on the results, the dual‐contrast method holds potential for imaging cartilage defects and overall cartilage condition of whole joints. When combined with standing CT, it could provide anaesthesia‐free evaluation of the health status of articular cartilage in horses, rapidly after injury before development of post‐traumatic OA.

## ETHICAL ANIMAL RESEARCH

The experimental model used in this study was approved by the Utrecht University Animal Experiments Committee and the Central Committee for Animal Experiments (permit AVD108002015307).

## INFORMED CONSENT

Not applicable.

## CONFLICT OF INTERESTS

No conflicts of interest have been declared.

## AUTHORSHIP

This original study was planned by M. Honkanen, A. Mohammadi, N. te Moller, R. van Weeren, R. Korhonen, J. Töyräs and J. Mäkelä. The bismuth nanoparticles were prepared by W. Xu in the lab of V‐P. Lehto. A. Mohammadi and B. Pouran conducted the CECT measurements at Utrecht University. N. te Moller and S. Plomp were responsible for sample collection and preparation. The digital densitometry measurements and analysis were conducted by A. Mohammadi and M. Ebrahimi. M. Honkanen analysed the CECT measurements and compiled all results. M. Honkanen, A. Mohammadi, J. Töyräs and J. Mäkelä participated in the evaluation and interpretation of the results. M. Honkanen prepared the manuscript with the help of co‐authors. All the authors have approved the final submitted version of the manuscript.

### PEER REVIEW

The peer review history for this article is available at https://publons.com/publon/10.1111/evj.13573.

## Data Availability

The data that support the findings of this study are available from the corresponding author upon reasonable request.
